# Colocutaneous Fistula Presenting as a Lateral Thigh Mass With Secondary Prosthetic Hip Infection

**DOI:** 10.7759/cureus.111056

**Published:** 2026-06-17

**Authors:** Jonathan Alverio Rivera, Sonal Kaushik, John Barnwell

**Affiliations:** 1 Surgery, University of Medicine and Health Sciences (UMHS), Basseterre, KNA; 2 Department of General Surgery, Detroit Medical Center Sinai Grace Hospital, Michigan, USA; 3 Department of General Surgery, Detroit Medical Center Huron Valley-Sinai Hospital, Michigan, USA

**Keywords:** colocutaneous fistula, fistulas, prosthetic joint, right thigh abscess, thigh abscess

## Abstract

An 83-year-old woman presented with progressive right lateral hip pain and a gradually enlarging lateral thigh mass in the setting of prior abdominal surgery and right total hip arthroplasty. No definitive bowel communication was identified, although evaluation was limited by artifact from the prosthesis. Given these findings, it was presumed to be an abscess. However, the proximity of the bowel to the mass raised concern for a possible hernia or fistulous process. The patient underwent incision and drainage of the right lateral thigh, and unexpectedly, a feculent material was found within the cavity, raising concern for a colocutaneous fistula with the involvement of the prosthetic joint. This case highlights the diagnostic challenge of enteric fistulas presenting in atypical extraperitoneal locations, such as the lateral thigh, and emphasizes the importance of considering enteric sources in deep soft tissue infections containing gas, even when imaging may be unclear.

## Introduction

Colocutaneous fistulas represent abnormal communications between the colon and skin and most commonly arise as complications of prior abdominal surgery, inflammatory bowel disease, malignancy, diverticulitis, or radiation therapy [1-3]. Typical presentations include persistent cutaneous drainage, localized infection, or sepsis near the abdominal wall [1,4]. A presentation involving the lateral thigh is rare and can make the diagnosis very challenging, particularly in patients with prior orthopedic hardware [2,5]. When infection involves a prosthetic joint, it becomes important to determine whether the source is primary to the joint or represents contiguous spread from another site, as management strategies differ. In this case report, we present a case of a colocutaneous fistula that initially presented as a lateral thigh mass with involvement of a prosthetic hip joint. Our purpose is to illustrate diagnostic pitfalls and the importance of multidisciplinary evaluation.

## Case presentation

An 83-year-old woman with a history of right total hip arthroplasty in 2021, asthma, chronic obstructive pulmonary disease, osteoarthritis, breast cancer status post mastectomy, prior small bowel resection, hysterectomy, and cholecystectomy presented to the emergency department with one week of progressive generalized weakness, poor oral intake, and worsening right lateral hip pain. She reported being in her usual state of health until June 2025, when she noticed a slowly enlarging mass over the right lateral hip. She was evaluated in the outpatient setting, where imaging reportedly suggested a benign soft-tissue mass, lipoma vs hernia, and no intervention was pursued due to lack of symptoms.

Approximately one week prior to presentation, she developed chills, diarrhea, ear pain, headache, and generalized malaise. During this period, she sustained a mechanical fall from bed, landing on her right side, after which she experienced increasing right hip pain and difficulty ambulating. She was evaluated by her primary care physician and started on antibiotics for presumed sinusitis, which she discontinued after several days due to lack of improvement.

Over the following days, her hip pain progressed, and she became increasingly weak with markedly reduced oral intake. On the day of presentation, she was unable to rise from a seated position, prompting family members to bring her to the emergency department. 

On arrival, she was tachycardic and hypotensive but afebrile. She appeared chronically ill and dehydrated. Cardiopulmonary examination revealed tachycardia. The abdomen was soft and nondistended. Examination of the right hip demonstrated a large, soft, compressible, tender mass over the lateral hip and buttock with pain on attempted abduction and limited mobility secondary to pain. No overlying erythema or warmth was initially noted. 

The laboratory and microbiological findings are summarized in Table 1.

**Table 1:** Laboratory and microbiological findings during hospitalization BUN: blood urea nitrogen, WBC: white blood cell, CRP: C-reactive protein

Parameter	Value (peak)	Reference range
Sodium (Na⁺)	127 mmol/L	135–145 mmol/L
Potassium (K⁺)	3.3 mmol/L	3.5–5.0 mmol/L
BUN	26 mg/dL	7–25 mg/dL
Creatinine	0.71 mg/dL	0.6–1.2 mg/dL
Albumin	2.4 g/dL	3.5–5.0 g/dL
WBC count	13.4 ×10⁹/L	4.0–11.0 ×10⁹/L
CRP	156.3 mg/L	<5.0 mg/L
Serum lactate	2.3 mmol/L	<2.0 mmol/L
Blood cultures	No growth	—
Abscess culture	*Klebsiella pneumoniae*; *Candida albicans*	—
Repeat cultures	No growth	—

On imaging, plain radiographs of the pelvis and right hip demonstrated subcutaneous gas over the proximal right thigh without fracture or prosthetic dislocation (Figure 1). CT of the abdomen, pelvis, and right lower extremity performed with oral contrast demonstrated a large peripherally enhancing gas-containing fluid collection posterolateral to the right hip, concerning for abscess formation, although no oral contrast was seen tracking into the cavity (Figures 2-6). MRI of the pelvis demonstrated a large rim‑enhancing collection extending toward the right hip prosthesis with an associated tract into the presacral pelvis (Figures 7-9). No definitive bowel communication was identified. 

**Figure 1:**
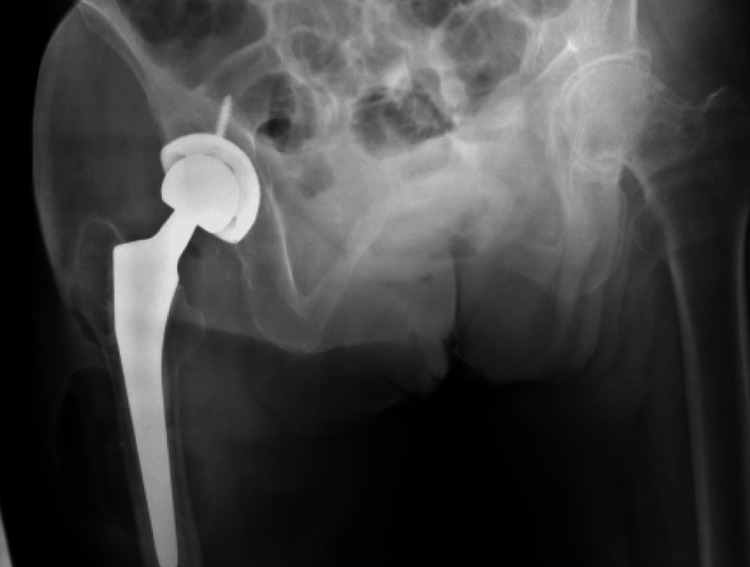
Plain radiograph of the right hip and pelvis showing subcutaneous gas over the proximal right thigh, no prosthetic dislocation.

**Figure 2:**
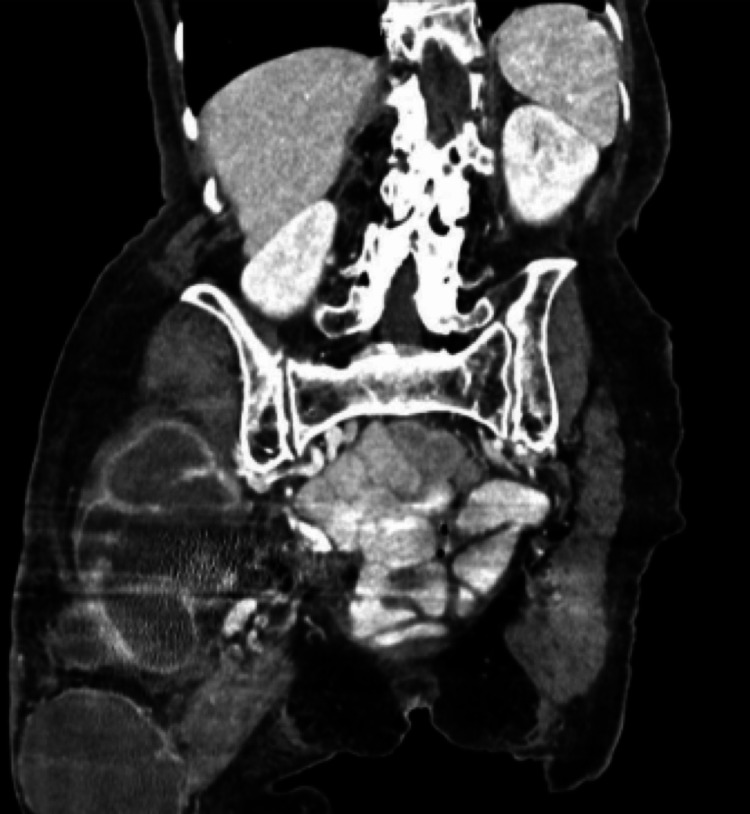
CT abdomen/pelvis coronal view showing gas-containing fluid collection posterolateral to the right hip.

**Figure 3:**
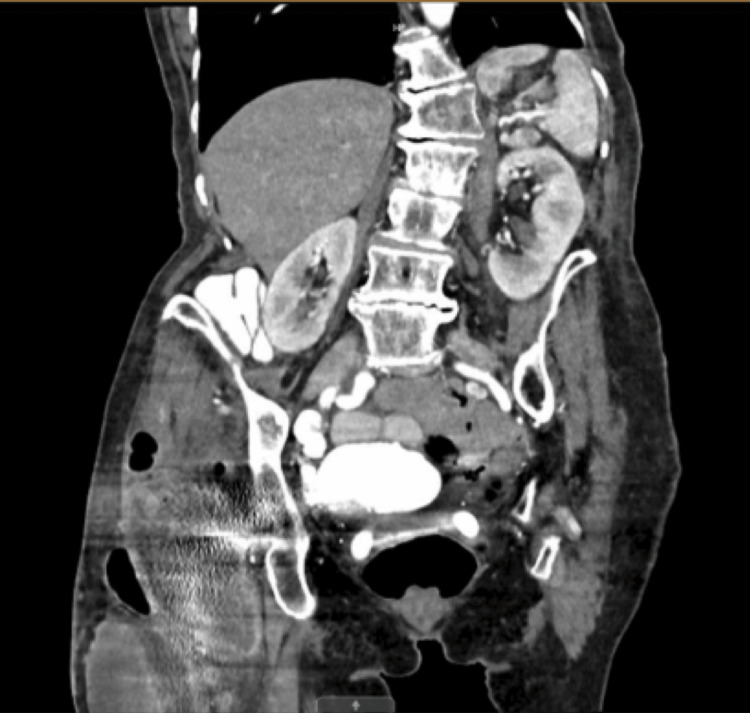
CT abdomen/pelvis coronal view (alternate slice) demonstrating extension of collection toward the right prosthesis toward the right prosthesis.

**Figure 4:**
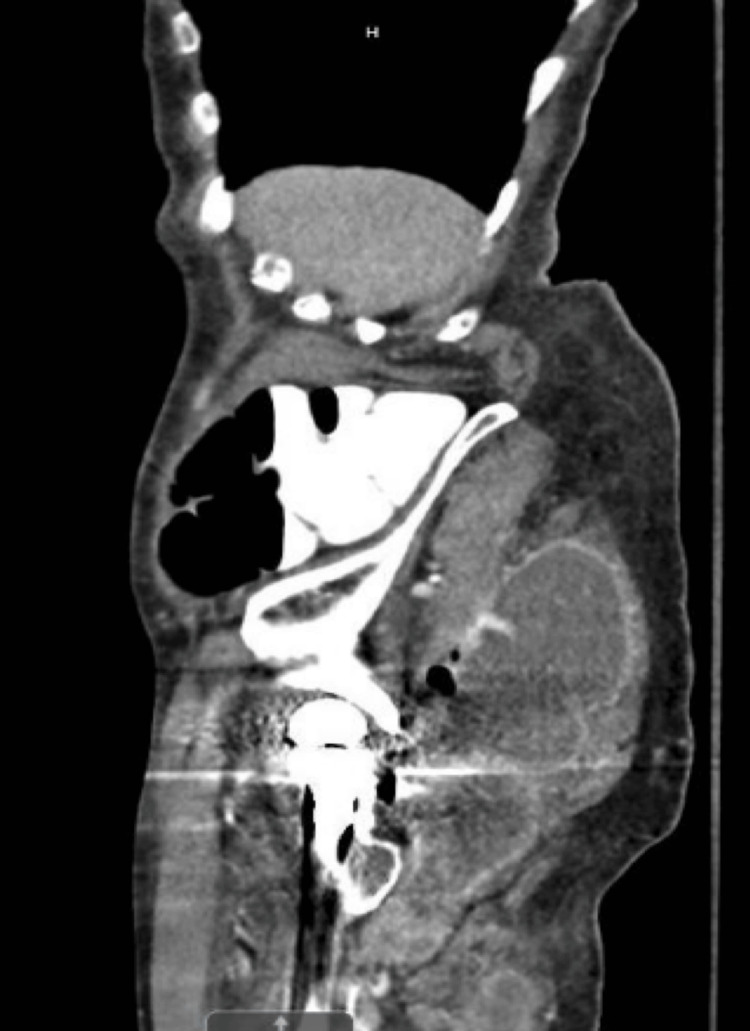
CT abdomen/pelvis sagittal view showing the tract extending from the presacral pelvis toward the right lateral thigh.

**Figure 5:**
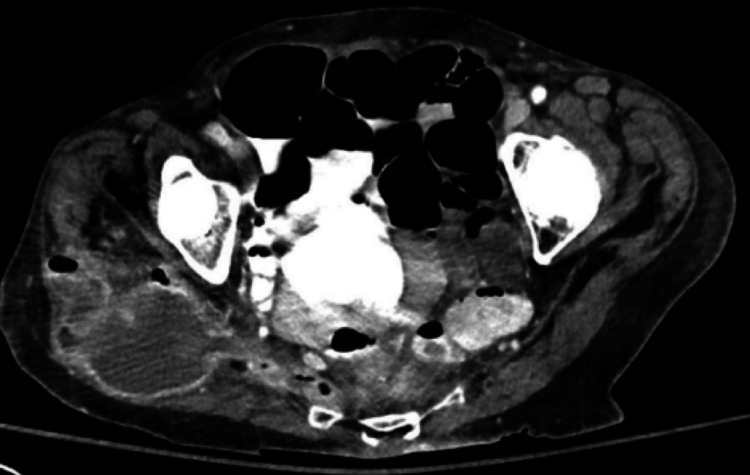
CT abdominal/pelvis axial view showing right-side rim-enhancing fluid collection with an internal gas locule.

**Figure 6:**
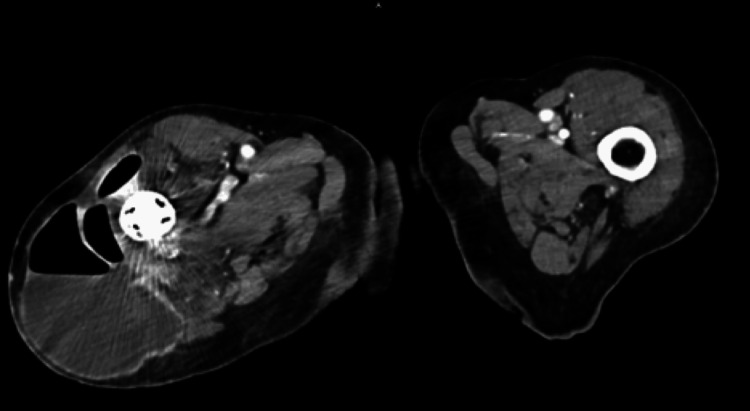
CT abdominal/pelvis axial view (inferior slice) showing proximity of collection to the right prosthetic hip.

**Figure 7:**
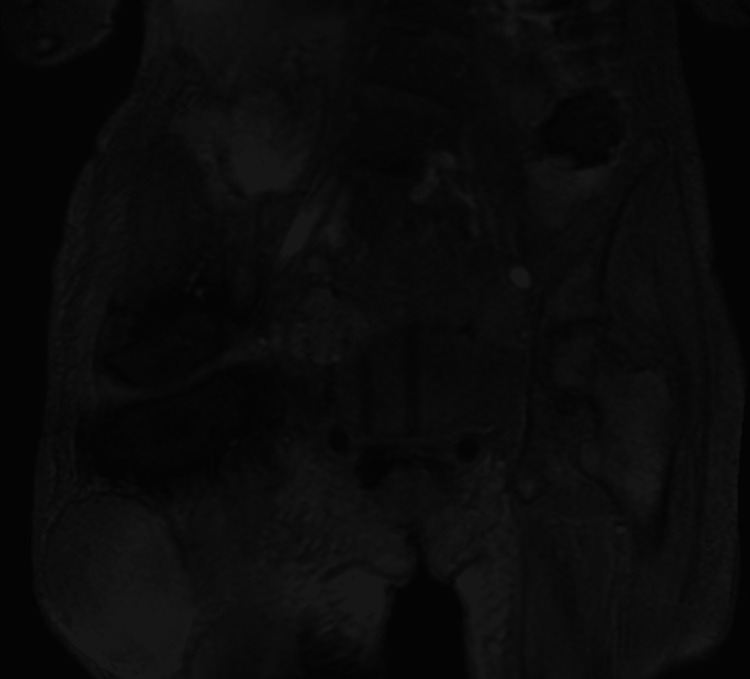
MRI pelvis coronal view showing right-side rim-enhancing collection and tract toward the presacral space.

**Figure 8:**
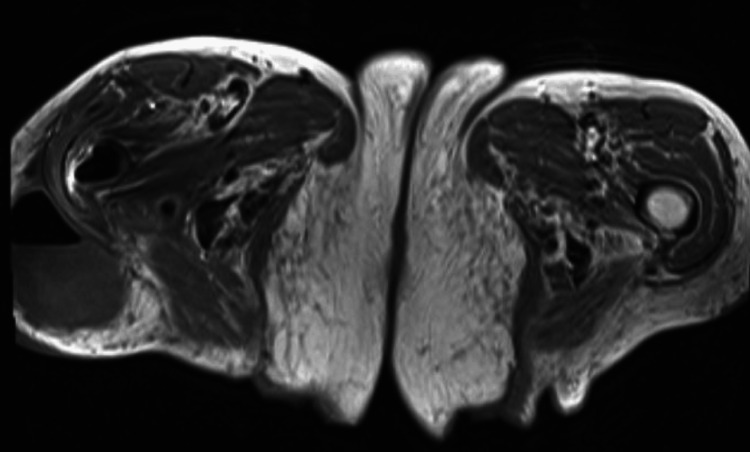
MRI pelvis axial view showing right-side rim-enhancing collection adjacent to the prosthesis.

**Figure 9:**
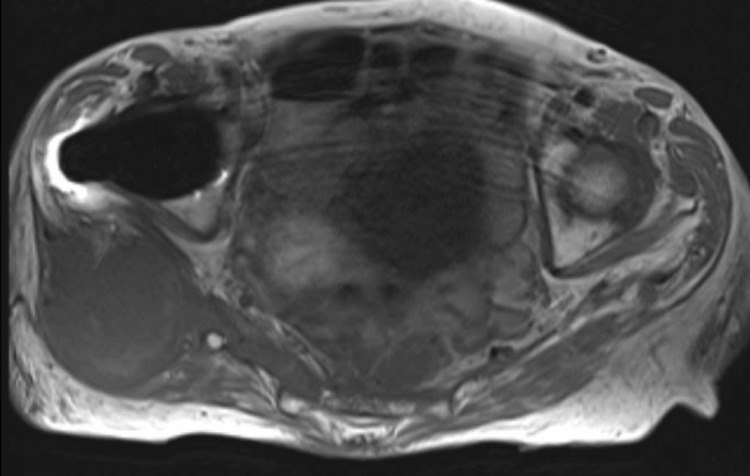
MRI pelvis axial image showing a rim‑enhancing fluid collection adjacent to the right hip prosthesis with extension toward the right lateral thigh.

Given these findings, the differential diagnosis included deep soft tissue abscess, femoral hernia with bowel involvement, colocutaneous fistula, and late prosthetic joint infection. The patient was managed with aggressive intravenous fluid resuscitation, electrolyte correction, and broad-spectrum antibiotics. Due to concern for deep infection and possible fistulous communication, she was taken to the operating room for examination under anesthesia and incision and drainage of the right hip mass. 

Intraoperatively, a large abscess within the thigh was identified and thoroughly drained and irrigated. Purulent material identical in appearance to the fluid within the abscess cavity was found on rectal exam under anesthesia, and it was removed from the cavity during rectal examination, raising strong concern for a colocutaneous fistula. No definitive bowel lumen was visualized within the thigh. The wound was left open and packed. Intraoperative abscess cultures grew Klebsiella pneumoniae and numerous Candida albicans.

Postoperatively, the patient was managed with broad-spectrum antibiotics, including anaerobic coverage and antifungal therapy. Due to severe protein-calorie malnutrition, total parenteral nutrition was initiated. Multidisciplinary consultations were obtained from colorectal surgery, orthopedic surgery, infectious disease, and critical care. Persistent feculent drainage from the right hip wound supported ongoing concern for a colocutaneous fistula. A CT scan with rectal contrast was planned for operative mapping, which would have guided a colectomy; however, the patient ultimately elected to pursue comfort‑focused care.

## Discussion

Fistulas are abnormal tunnels that connect two structures that do not normally connect. Colocutaneous fistulas are pathological connections between the colon and the skin. The most common causes of colocutaneous fistulas are abdominal surgery, diverticulitis, Crohn’s disease, gastrointestinal inflammation, colorectal cancer, and radiation therapy [1,2]. When these fistulous tracts become contaminated with enteric contents, they can seed infection in the surrounding soft tissues, leading to abscess formation [3].

Abscesses form when the immune system attempts to contain infection by creating a walled-off cavity composed of inflammatory cells, necrotic debris, and bacteria [3]. In rare cases where colocutaneous fistulas extend into the thigh, the anatomical location of the resulting abscess often reflects the anatomical origin of the fistula and provides diagnostic guidance, with right-sided thigh abscesses raising suspicion for a cecum or appendiceal origin, whereas left-sided abscesses suggest sigmoid or rectal disease [4].

Several unusual presentations have been described in the literature. Huda and Pandya reported a case of a left thigh abscess caused by a colocutaneous fistula in a patient with Crohn’s disease, in whom CT imaging demonstrated a tract extending from the rectum into the thigh [1]. Koroglu et al. described a patient who developed bilateral thigh abscesses nine years after colectomy for colorectal cancer, ultimately found to have an enterocutaneous fistula requiring fistulotomy and radical debridement [4]. Other reports highlight alternative etiologies, including a case of necrotizing fasciitis originating from emphysematous pyelonephritis associated with a staghorn calculus, which extended into the thigh and required urgent surgical debridement [5], as well as a case of sigmoid diverticulitis presenting as a contralateral thigh abscess [6].

Most reported cases involved patients with known gastrointestinal pathology, such as inflammatory bowel disease, colorectal cancer, prior abdominal surgery, or infections, that promote the formation of a fistula to the thigh [2]. In these cases, imaging typically demonstrates a fistulous connection before operative intervention, and the past medical history provides some evidence pointing toward the gut as the source. Our case is unique in that the patient lacked a clear history of active gastrointestinal disease, and preoperative imaging did not definitively identify a fistulous tract. This created a diagnostic challenge, particularly given the proximity of the collection to a prosthetic hip joint, which broadened the differential diagnosis to include deep soft-tissue abscess, femoral hernia, and late prosthetic joint infection.

Ultimately, surgical exploration was required to establish the diagnosis. The presence of feculent material within the abscess cavity confirmed an enteric source despite the absence of a clearly visualized bowel lumen. This case underscores the importance of maintaining a broad differential diagnosis when evaluating deep soft-tissue infections containing gas, especially in atypical anatomical locations, and highlights the value of early multidisciplinary involvement when imaging and clinical findings do not align.

## Conclusions

Colocutaneous fistulas may present in atypical extraperitoneal locations, creating diagnostic uncertainty when gastrointestinal symptoms are absent, and imaging does not clearly demonstrate a tract. In this case, the chronic lateral thigh mass evolved into a gas‑containing deep infection whose enteric origin became evident only at operative exploration, reflecting known limitations of cross‑sectional imaging in detecting small or complex fistulous pathways. The intraoperative discovery of a feculent material confirmed the diagnosis and highlights the need to consider an enteric source when gas is present in deep soft tissues, even in patients without known gastrointestinal disease. Early surgical evaluation and multidisciplinary collaboration are essential when clinical progression and radiographic findings diverge, particularly when a prosthetic joint is at risk, as timely intervention is critical to reducing morbidity.
